# Endothelial Glycocalyx of Peritubular Capillaries in Experimental Diabetic Nephropathy: A Target of ACE Inhibitor-Induced Kidney Microvascular Protection

**DOI:** 10.3390/ijms242216543

**Published:** 2023-11-20

**Authors:** Monica Locatelli, Daniela Rottoli, Rayan Mahmoud, Mauro Abbate, Daniela Corna, Domenico Cerullo, Susanna Tomasoni, Giuseppe Remuzzi, Carlamaria Zoja, Ariela Benigni, Daniela Macconi

**Affiliations:** Istituto di Ricerche Farmacologiche Mario Negri IRCCS, Centro Anna Maria Astori, Science and Technology Park Kilometro Rosso, 24126 Bergamo, Italy; monica.locatelli@marionegri.it (M.L.); daniela.rottoli@marionegri.it (D.R.); rayan.mahmoud@marionegri.it (R.M.); mauro.abbate@marionegri.it (M.A.); daniela.corna@marionegri.it (D.C.); domenico.cerullo@marionegri.it (D.C.); susanna.tomasoni@marionegri.it (S.T.); giuseppe.remuzzi@marionegri.it (G.R.); carlamaria.zoja@marionegri.it (C.Z.); daniela.macconi@marionegri.it (D.M.)

**Keywords:** diabetic nephropathy, peritubular capillary, endothelial glycocalyx, angiotensin-converting enzyme inhibitor

## Abstract

Peritubular capillary rarefaction is a recurrent aspect of progressive nephropathies. We previously found that peritubular capillary density was reduced in BTBR *ob*/*ob* mice with type 2 diabetic nephropathy. In this model, we searched for abnormalities in the ultrastructure of peritubular capillaries, with a specific focus on the endothelial glycocalyx, and evaluated the impact of treatment with an angiotensin-converting enzyme inhibitor (ACEi). Mice were intracardially perfused with lanthanum to visualise the glycocalyx. Transmission electron microscopy analysis revealed endothelial cell abnormalities and basement membrane thickening in the peritubular capillaries of BTBR *ob*/*ob* mice compared to wild-type mice. Remodelling and focal loss of glycocalyx was observed in lanthanum-stained diabetic kidneys, associated with a reduction in glycocalyx components, including sialic acids, as detected through specific lectins. ACEi treatment preserved the endothelial glycocalyx and attenuated the ultrastructural abnormalities of peritubular capillaries. In diabetic mice, peritubular capillary damage was associated with an enhanced tubular expression of heparanase, which degrades heparan sulfate residues of the glycocalyx. Heparanase was also detected in renal interstitial macrophages that expressed tumor necrosis factor-α. All these abnormalities were mitigated by ACEi. Our findings suggest that, in experimental diabetic nephropathy, preserving the endothelial glycocalyx is important in order to protect peritubular capillaries from damage and loss.

## 1. Introduction

Peritubular capillary rarefaction is a common event in the progression of chronic kidney disease (CKD) with different aetiologies and is associated with impaired renal function and tubular interstitial injury [1,2,3,4]. In CKD patients, peritubular capillary rarefaction better reflects the glomerular filtration rate (GFR) decline than other features of progressive nephropathy, such as tubular atrophy, interstitial fibrosis, and inflammation [5]. Consistent with this, the loss of peritubular capillaries in response to an acute kidney injury strongly influences the disease outcome toward the CKD progression, because it is closely correlated with a reduced renal function [6]. Furthermore, the evidence that peritubular capillary rarefaction precedes the development of tubulointerstitial fibrosis, as demonstrated by longitudinal studies in experimental animals [7], suggests that it plays a crucial role in regulating and driving the CKD progression.

Experimental studies in different models of progressive CKD with fibrosis, including unilateral ureteral obstruction and ischaemia-reperfusion injury, have shown a close link between the loss of the structural and functional integrity of peritubular capillaries and their rarefaction [8]. Abnormalities were independent of the primary insult and ultrastructural changes in the peritubular capillary endothelium manifested early during the disease. The in vivo assessment of microvascular permeability revealed hypoperfusion and an increased leakiness in peritubular capillaries of the fibrotic kidneys, compared to capillaries of healthy kidneys [8]. Moreover, a recent investigation in the same models of renal fibrosis demonstrated that peritubular capillaries in diseased kidneys exhibited a reduction in the thickness and density of the endothelial glycocalyx [9]. The latter is a negatively charged network of proteoglycans, glycosaminoglycans, glycoproteins, and glycolipids that covers the luminal surface of vascular endothelial cells, and has pleiotropic functions, including the regulation of vascular permeability, inflammation, and mechanotransduction [10,11,12,13].

A reduction in peritubular capillary density has also been detected in kidneys from both animals and patients with diabetic nephropathy (DN) [14,15,16]. Experimental studies in rats with type 1 diabetes have demonstrated that peritubular capillaries are leaky, causing albumin to extravasate in the renal interstitium, which has been suggested to be responsible for the increase in renal interstitial volume and pressure, thus ultimately affecting the renal function [17]. Whether alterations in the endothelial glycocalyx could be associated with the loss of structural integrity of peritubular capillaries, possibly contributing to microvascular dysfunction and damage, has not been investigated in experimental diabetes. In BTBR *ob*/*ob* mice, a model of type 2 DN, we demonstrated that the treatment with an angiotensin-converting enzyme inhibitor (ACEi) antagonized peritubular capillary rarefaction [15]. However, no studies have addressed whether a drug treatment to mitigate peritubular capillary rarefaction could afford favourable effects on the peritubular capillary endothelial glycocalyx.

In the present study, we aimed (1) to characterise the ultrastructure of peritubular capillaries, including the endothelial glycocalyx, in diabetic BTBR *ob*/*ob* mice through transmission electron microscopy (TEM) analysis; (2) to investigate whether ACEi treatment could maintain the structural integrity of peritubular capillaries in this model; and (3) to elucidate whether the ACEi treatment preserved the peritubular capillary endothelial glycocalyx.

## 2. Results

### 2.1. Laboratory, Systemic, and Renal Function Parameters

Table 1 displays the laboratory, systemic, and renal function parameters that were assessed in mice, both nondiabetic and diabetic, when they reached 18 weeks of age. BTBR *ob*/*ob* mice administered a vehicle were obese and exhibited high blood glucose levels compared with BTBR wild-type (WT) mice. There were no differences in body weight between the groups of diabetic mice, and hyperglycaemia was not affected by the treatment with the ACEi, lisinopril. Consistent with previous studies [15,18,19], BTBR *ob*/*ob* mice administered a vehicle did not develop hypertension. Lisinopril significantly reduced the systolic blood pressure in diabetic mice. To evaluate the renal function, the glomerular filtration rate (GFR) was measured through iohexol plasma clearance. Eighteen-week-old diabetic mice administered a vehicle exhibited a significant increase in the GFR compared to WT mice, which was slightly, though not significantly, reduced by lisinopril. Diabetic mice exhibited albuminuria, which was evaluated using the urinary albumin-to-creatinine ratio (uACR) and was significantly reduced by lisinopril.

### 2.2. Kidneys of Diabetic Mice Exhibit Peritubular Capillary Rarefaction, Which Is Limited by ACEi Treatment

**Figure 1:**
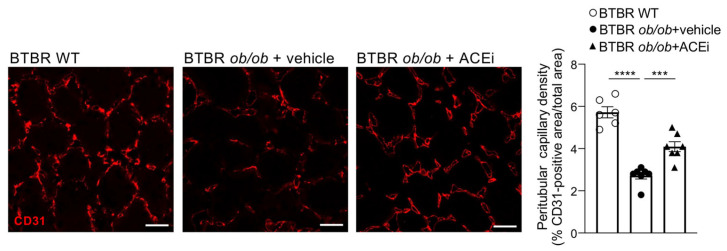
Peritubular capillary rarefaction in BTBR *ob*/*ob* mice is limited by lisinopril treatment. Representative images of CD31 expression in the interstitium of the cortex of BTBR WT and BTBR *ob*/*ob* mice received vehicle or treated with the ACEi lisinopril. Quantification of peritubular capillary density expressed as % of CD31-positive area/total area. Data are mean ± SEM. *** *p* < 0.001, **** *p* < 0.0001. Bar = 20 µm.

BTBR *ob*/*ob* diabetic mice exhibited a reduced peritubular capillary density in the cortex compared to WT mice, as demonstrated by a reduced CD31 staining. Consistent with previous findings [15], treatment with ACEi significantly attenuated the peritubular capillary loss (Figure 1).

### 2.3. The Ultrastructure of Peritubular Capillaries Is Altered in Diabetic Mice and Ameliorated by ACEi Treatment

**Figure 2:**
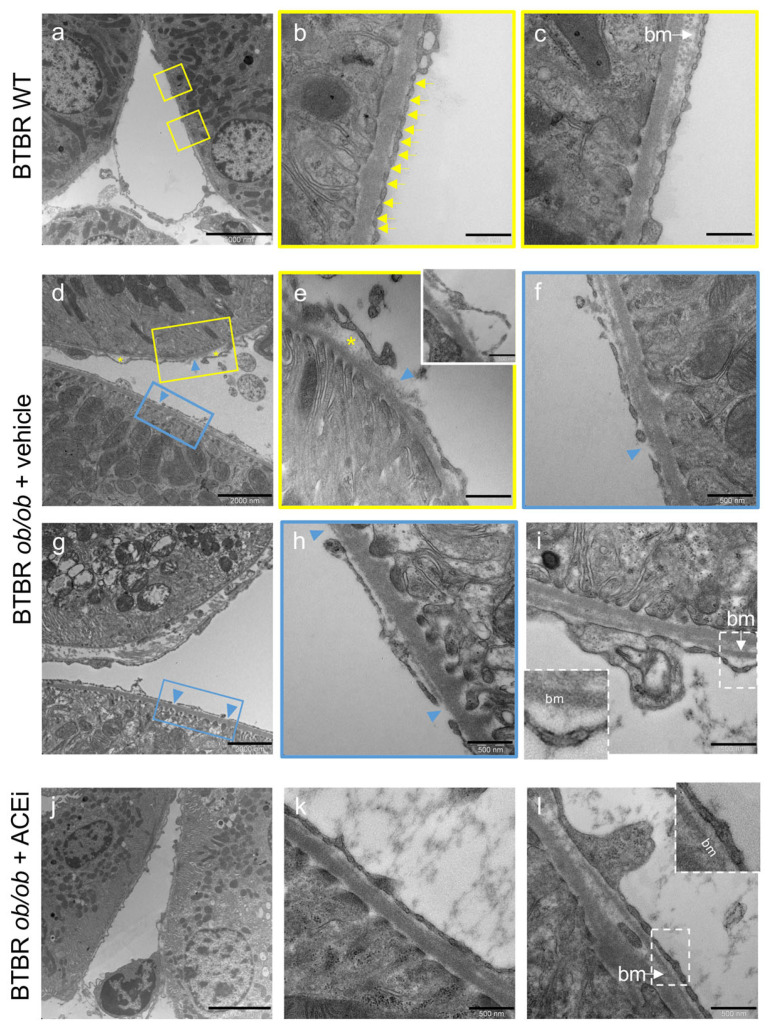
The peritubular capillary ultrastructure is altered in BTBR *ob*/*ob* mice and ameliorated by ACEi treatment. Representative TEM microscopy images of peritubular capillary ultrastructure in BTBR WT mice and in diabetic BTBR *ob*/*ob* mice received vehicle or treated with the ACEi lisinopril. (**a**–**c**) Overview and details of a peritubular capillary in BTBR WT: normally fenestrated endothelium with diaphragms (arrows, **b**) and basement membrane (**c**). (**d**–**i**) Peritubular capillaries from vehicle treated BTBR *ob*/*ob* mice showing focal separation and delamination of endothelial cell from the basement membrane (asterisks, (**d**,**e**); insert in (**e**)), with lucent areas and amorphous material, denuded basement membrane (arrowheads, (**d**–**h**)) or foci of basement membrane thickening (bm, (**i**)). (**j**–**l**) Peritubular capillary from ACEi-treated mouse with normal-appearing structure (**j**). Images at high magnification from other capillaries showing endothelial cells covering their basement membrane ((**k**,**l**), inset). Bar = 5000 nm (**a**,**j**); 2000 nm (**d**,**g**) and 500 nm (**b**,**c**,**e**,**f**,**h**,**i**,**k**,**l**).

TEM analysis was performed to assess whether ultrastructural abnormalities could be detected in peritubular capillaries of BTBR *ob*/*ob* mice. In BTBR WT mice, the peritubular capillary appeared as mainly flattened, fenestrated endothelium with diaphragms, and a basement membrane layer beneath the endothelial cell (Figure 2a–c). In diabetic mice, peritubular capillaries exhibited focal and irregularly distributed ultrastructural abnormalities that were absent in WT mice. Specifically, the endothelial cell layer had the features of delamination (Figure 2d,e, asterisks; insert in e) and denudation of the underlying basement membrane (Figure 2d,h arrowheads). Notably, multiple pathological changes were present in the same capillary, with a partial loss of normal diaphragmed fenestration (Figure 2d–f). Another feature was the presence of a thickened basement membrane compared to the control group (Figure 2i vs. Figure 2c). We estimated the percentage of peritubular capillaries that were affected by these ultrastructural changes in diabetic mice and investigated whether this percentage was changed by ACEi treatment. In BTBR *ob*/*ob* mice administered a vehicle, the percentage of peritubular capillaries that exhibited an endothelial cell delamination and a denuded basement membrane averaged 18 ± 9% and 27 ± 9%, respectively. Lisinopril-treated diabetic mice exhibited a lower extent of peritubular capillary damage (Figure 2i–l), with an endothelial cell delamination detected in 2 ± 2% of examined peritubular capillaries, while a denuded basement membrane was detected in 9 ± 5%. The thickening of the basement membrane was found in 72 ± 11% of peritubular capillaries in diabetic mice that received a vehicle, which was decreased to 42 ± 9% by ACEi treatment.

### 2.4. The Endothelial Glycocalyx of Peritubular Capillaries Is Altered in Diabetic Mice and Preserved by ACEi Treatment

**Figure 3:**
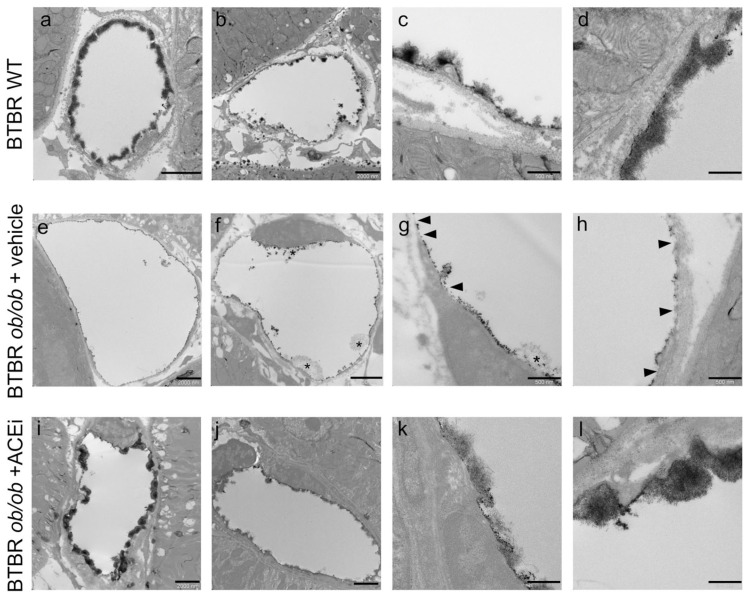
The endothelial glycocalyx of peritubular capillaries is altered in diabetic mice and preserved by ACEi treatment. Representative TEM images of lanthanum-stained endothelial glycocalyx of peritubular capillaries in BTBR WT mice and in diabetic BTBR *ob*/*ob* mice received vehicle or treated with the ACEi lisinopril. (**a**–**d**) The endothelial glycocalyx of peritubular capillaries in BTBR WT showing a dense layer covering the luminal surface of the capillary with a moss-like pattern well visible at high magnification (**c**,**d**). (**e**–**h**) The endothelial glycocalyx in diabetic mice appears granular and discontinuous with areas of loss or reduced coverage ((**g**,**h**) arrowheads) and the occasional presence of loose network ((**f**,**g**) asterisks). (**i**–**l**) The endothelial glycocalyx is maintained by lisinopril treatment showing a pattern similar to that observed in WT mice. Bar = 2000 nm (**a**,**b**,**e**,**f**,**i**,**j**) and 500 nm (**c**,**d**,**g**,**h**,**k**,**l**).

The endothelial glycocalyx of peritubular capillaries was visualised through TEM analysis of mouse kidneys after the intracardiac perfusion of lanthanum solution. BTBR WT control mice revealed a marked lanthanum endothelial staining, which was mostly continuous (Figure 3a), although in some capillaries, it was less regular (Figure 3b), indicating a variability among healthy peritubular capillaries, as recently reported in mice [9]. A moss-like pattern covering the luminal surface was observed (Figure 3c,d). Unlike in controls, lanthanum staining of peritubular capillaries of BTBR *ob*/*ob* mice administered a vehicle exhibited a granular electron dense layer lining the luminal surface of the endothelium (Figure 3e,f), with a linear, discontinuous pattern in the majority of examined capillaries. There was a focal loss of membrane-attached glycocalyx or a reduced luminal coverage where the staining appeared to be sparser (Figure 3g,h, arrowheads). The occasional presence of areas with a loose network was also observed (Figure 3f,g, asterisks). In all ACEi-treated diabetic mice but one, the endothelial glycocalyx of peritubular capillaries appeared as a dense, moss-like layer that was continuous or interspersed with linear staining, resembling the pattern found in control mice.

**Figure 4:**
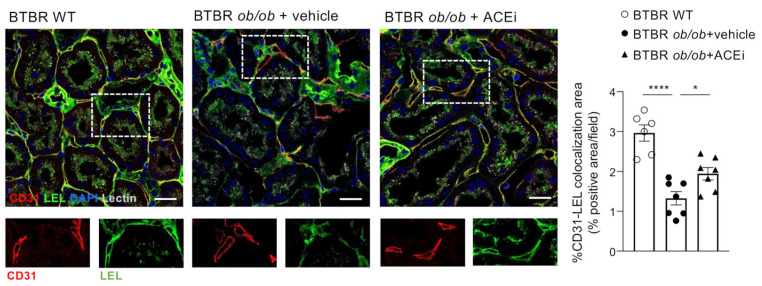
Remodeling of endothelial glycocalyx in peritubular capillaries of diabetic mice, assessed by staining with LEL is prevented by ACEi treatment. Representative images of immunofluorescence staining for peritubular capillaries (CD31, in red), endothelial glycocalyx (LEL, in green) cell nuclei (DAPI, in blue) and renal stuctures (lectin, pseudocolor) in the renal interstitium of BTBR WT mice and BTBR *ob*/*ob* mice received vehicle or treated with the ACEi lisinopril. Colocalization of CD31 and LEL signals appears in yellow and its quantification is expressed as % of positive area/field. Insets show CD31-positive cells that also express LEL binding sites in BTBR WT mice and in ACEi–treated diabetic mice, while they are negative for LEL in diabetic mice received vehicle. Bar = 20 μm. Data are mean ± SEM. * *p* < 0.05, **** *p* < 0.0001.

The endothelial glycocalyx was further analysed using the immunofluorescence of lycopersicon esculentum lectin (LEL) staining, which labels 1,3 *N*-acetylglucosamine and binds to both complex type and high-mannose type *N*-glycans glycoproteins [20,21]. Double staining experiments for LEL-binding sites and CD31 showed a significant reduction in the lectin-associated staining in the peritubular capillaries of BTBR *ob*/*ob* mice administered a vehicle compared to WT mice (Figure 4). Treatment with lisinopril improved the LEL staining of peritubular endothelial glycocalyx.

### 2.5. Staining for Sialic Acid Is Reduced in Peritubular Capillaries of Diabetic Mice and Is Improved by ACEi Treatment

**Figure 5:**
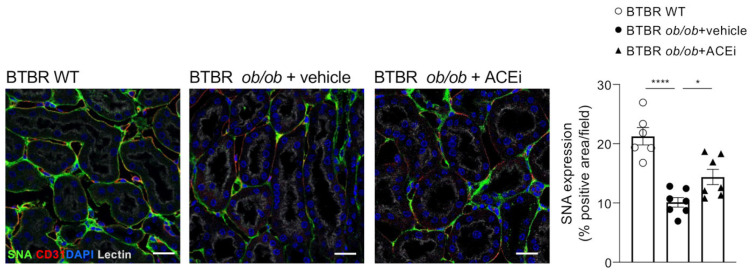
Staining for sialic acid is reduced in peritubular capillaries of diabetic mice and improved by ACEi treatment. Representative images and quantification of sialic acid (SNA, green) immunofluorescence staining in the renal interstitium of BTBR WT mice and diabetic BTBR *ob*/*ob* mice received vehicle or treated with the ACEi lisinopril. Data are mean ± SEM. * *p* < 0.05, **** *p* < 0.0001. Cell nuclei (DAPI, in blue), renal structures (lectin, pseudocolor). Bar = 20 µm.

The study and characterisation of the anionic sites at the luminal surface of peritubular capillaries has demonstrated that neuraminic acid is the predominant anionic site in the glycocalyx, while heparan sulfate (HS) mainly localises at the fenestral diaphragm [22]. Based on this evidence, we investigated whether sialic acids, the neuraminic acid derivatives, were altered in the peritubular capillaries of diabetic mice. Immunofluorescence analysis using sambucus nigra lectin (SNA), which binds preferentially to sialic acid attached to terminal galactose with α-2,6 and, to a lesser degree, α-2,3 linkage, showed a reduced staining in the peritubular capillaries of BTBR *ob*/*ob* mice administered a vehicle compared to WT mice. ACEi treatment improved the SNA staining (Figure 5).

### 2.6. Interstitial Heparanase 1 Is Overexpressed in Diabetic Mice and Reduced by ACEi Treatment

**Figure 6:**
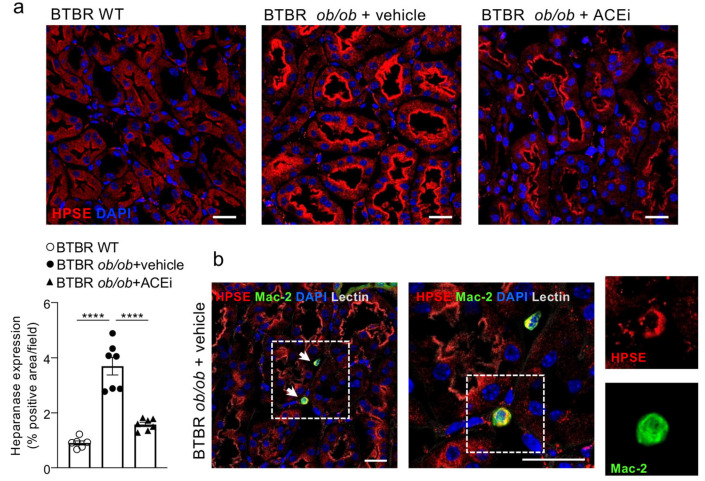
Lisinopril reduces interstitial heparanase overexpression in diabetic mice. (**a**) Representative images and quantification of HPSE expression (in red) in cortical tubules of BTBR WT mice and diabetic BTBR *ob*/*ob* mice received vehicle or treated with the ACEi lisinopril. A positive staining is observed in the brush border of proximal tubules. Data are mean ± SEM. **** *p* < 0.0001. Bar = 20 µm. (**b**) HPSE (in red) is detected in monocyte/macrophage (Mac-2, in green) interstitial infiltrates (arrows) in diabetic mice. Boxes show merge and single stainings of HPSE and Mac-2 in one interstitial infiltrate at high magnification. Cell nuclei (DAPI, in blue), renal structures (lectin, pseudocolor). Bar = 20 μm.

Heparan sulfate is a negatively charged glycosaminoglycan that attaches to a core protein of distinct proteoglycans, such as syndecans and glypicans, forming heparan sulfate proteoglycans, the most abundant proteoglycans present in the vascular glycocalyx [10]. Since the HS degradation is regulated by heparanase 1 (HPSE1) [23], which plays a crucial role in the development of DN, we analysed the enzyme expression in the renal interstitium of nondiabetic and diabetic mice. As shown in Figure 6a, HPSE1 was mildly expressed in the proximal tubules of the renal cortex of BTBR WT mice, while it was markedly increased in BTBR *ob*/*ob* mice administered a vehicle, mostly localised in the brush border. Heparanase was also detected in renal interstitial macrophages in diabetic mice, as demonstrated by the presence of Mac-2 and HPSE1 double-positive inflammatory cells (Figure 6b). Lisinopril-treated diabetic mice exhibited a significantly lower HPSE1 expression in renal proximal tubular epithelial cells (Figure 6a).

### 2.7. ACEi Treatment Attenuates Renal Interstitial Inflammation in Diabetic Mice

Inflammation is currently accepted as an important player in the onset and progression of DN [24], with macrophages contributing to the progressive renal injury [25].

**Figure 7:**
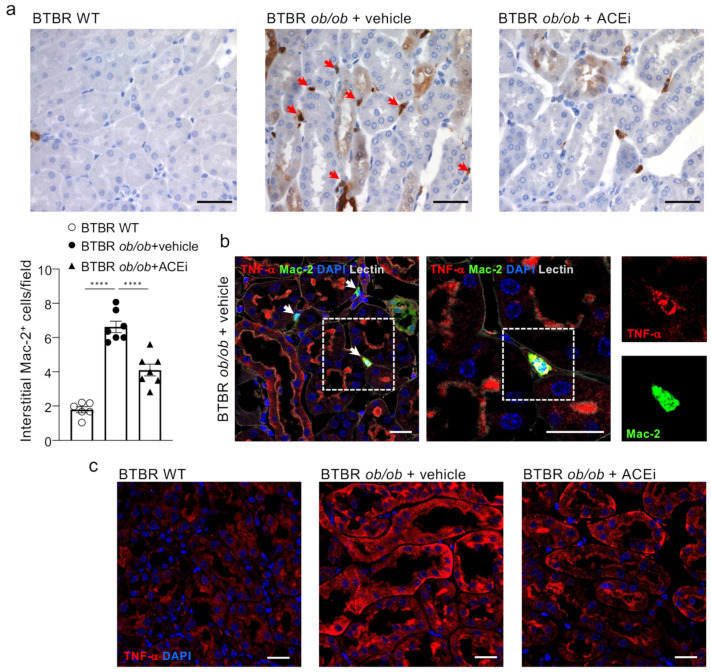
Lisinopril attenuates interstitial inflammation in diabetic mice. (**a**) Representative images and quantification of Mac-2–positive monocytes/macrophages in the renal interstitium of BTBR WT mice and diabetic BTBR *ob*/*ob* mice given vehicle or treated with the ACEi lisinopril (arrows). Data are mean ± SEM. **** *p* < 0.0001. Bar = 50 µm. (**b**) TNF-α (in red) is detected in monocyte/macrophage (Mac-2, in green) interstitial infiltrates (arrows) in diabetic mice. Boxes show merge and single stainings of TNF-α and Mac-2 in one interstitial infiltrate at high magnification. Cell nuclei (DAPI, in blue), renal structures (lectin, pseudocolor). Bar = 20 μm. (**c**) Representative images of immunofluorescence staining for TNF-α (in red) expressed in cortical tubules of BTBR WT mice and BTBR *ob*/*ob* mice received vehicle or treated with the ACEi lisinopril. Cell nuclei (DAPI, in blue). Bar = 20 μm.

Concomitant with peritubular capillary damage and loss, a marked accumulation of inflammatory Mac-2-positive monocytes/macrophages was found in the renal interstitium of BTBR *ob*/*ob* mice administered a vehicle, compared to BTBR WT mice (Figure 7a). Notably, macrophages in diabetic animals expressed tumor necrosis factor-α (TNF-α), which was also increased in the cortical tubules (Figure 7b,c). ACEi treatment lessened the renal inflammation in diabetic mice by significantly reducing infiltrating inflammatory cells and lowering tubular TNF-α expression (Figure 7a,c).

## 3. Discussion

The present study shows that BTBR *ob*/*ob* mice with type 2 DN manifest peritubular capillary rarefaction in association with the marked alteration or loss of the endothelial glycocalyx and ultrastructural changes, characterised by an endothelial cell delamination and a denuded and thick basement membrane. It also shows the role that renal tubules and interstitial inflammatory cells play as a source of mediators that are potentially responsible for peritubular capillary damage. Finally, the study highlights that an approach that acts to limit peritubular capillary rarefaction, namely, ACEi treatment, preserves the endothelial glycocalyx on peritubular capillaries.

Our findings on the loss of peritubular capillaries in BTBR *ob*/*ob* mice that exhibit glomerular hyperfiltration are in line with previous observations of peritubular capillary rarefaction in rats with type 1 DN [14] and indicate that peritubular capillary damage precedes the renal impairment that occurs in the late stage of the disease [18].

Here, we describe, in experimental type 2 DN, the presence of ultrastructural abnormalities in the peritubular capillary endothelium, along with the initial thickening of the basement membrane. So far, changes in the ultrastructure of peritubular capillaries have been reported mainly in the mouse models of CKD with fibrosis [8] and in rats with progressive glomerulonephritis [26] as well as in CKD patients with interstitial nephritis or ANCA-associated glomerulonephritis [8]. Areas of denuded basement membrane and endothelial cell delamination from the basement membrane that we found in the diabetic kidneys are features that are also observed in the injured peritubular capillary endothelium of mice with the genetic ablation of glioma-associated oncogene-positive pericytes, whose loss contributed to microvessel destabilisation and subsequent rarefaction [27]. Moreover, the increase in the thickness of the peritubular capillary basement membrane in BTBR *ob*/*ob* mice is consistent with morphological changes observed in human DN [28] and is a common alteration found in other experimental CKD models [8].

The most interesting and novel finding of the present study is the demonstration that the endothelial glycocalyx of peritubular capillaries is altered in experimental type 2 DN. Visualising the endothelial glycocalyx through the TEM analysis of lanthanum staining revealed a different morphological pattern and loss in BTBR *ob*/*ob* mice administered a vehicle compared to BTBR WT mice. In addition, the reduced lycopersicon esculentum lectin (LEL) staining indicates changes in the composition and remodelling of the glycocalyx that occurred in the peritubular capillaries of diabetic mice. This observation is in line with the earlier evidence of reduced LEL staining of the endothelial glycocalyx of peritubular capillaries in murine and human fibrotic kidneys [9].

Ultrastructural studies in rats have shown that, in healthy conditions, the peritubular capillary endothelial glycocalyx covers both the luminal surface of the endothelial cells and the fenestral diaphragms [29]. Evidence of the presence of anionic sites, such as neuraminic acid and HS in the glycocalyx and at the fenestral diaphragms of peritubular capillaries, indicates that the luminal surface of these cells can act as a charge-selective barrier [22]. Neuraminic acid and its derivatives, sialic acids, are components of polysaccharide chains in glycoproteins, including platelet endothelial adhesion molecule (PECAM/CD31), and regulate their biological functions [30]. Particularly, α2,6-sialic acid terminally bound to galactose of the *N*-glycans of PECAM/CD31 is necessary for protein retention on the cell surface, favouring PECAM-PECAM homophilic interactions, which regulate endothelial cell permeability, mechanotransduction, anti-apoptotic signalling, and angiogenesis [31,32,33,34]. Moreover, there is evidence that sialic acids in the endothelial glycocalyx play an important role in regulating microvessel permeability, as shown by changes in albumin permeability across rat mesenteric miocrovessels evaluated before and after the cleavage of terminal sialic acids using perfusion with neuraminidase [35].

Our finding that α2,6-sialic acid was reduced in peritubular capillaries in BTBR *ob*/*ob* mice administered a vehicle demonstrates that the loss of anionic components of peritubular endothelial glycocalyx could be involved in peritubular capillary dysfunction in DN.

There is also evidence that the enzymatic degradation of HS in peritubular capillaries is associated with albumin extravasation [22], which could result in changes in peritubular capillary oncotic pressure, influencing the capillary and renal tubule reabsorption of water and solutes [36]. Heparan sulfate side chains are degraded by HPSE1 [23]. The induction of HPSE1 in the glomerular podocytes of diabetic mice led to the disruption of the endothelial glycocalyx in glomerular capillaries, ultimately causing the impairment of the glomerular filtration barrier [37,38]. The evidence that HPSE1 is overexpressed in renal tubules of patients with DN [39,40] prompted us to investigate whether these cells may be a source of the enzyme in diabetic mice. We found that HPSE1 was indeed overexpressed in the renal proximal tubules of BTBR *ob*/*ob* mice. Consistent with in vitro studies showing that albumin and advanced glycated end products can trigger the induction of HPSE in proximal tubular cells [41], it is tempting to speculate that, under diabetic conditions, HPSE1 released from distressed tubules could contribute, in a paracrine manner, to the degradation of HS in peritubular capillaries.

On the other hand, the HPSE of epithelial origin has been reported to modulate macrophage responses in chronic inflammation, as documented in experimental colitis [42]. Similarly, HPSE enhanced the macrophage activation induced by a diabetic milieu, leading to the release of inflammatory cytokines, including TNF-α, thus sustaining inflammation [43,44,45]. Our finding that the macrophages infiltrating the renal interstitial parenchyma of diabetic mice expressed both HPSE1 and TNF-α suggests a possible functional link between HPSE and the pro-inflammatory response of interstitial macrophages in DN. Importantly, TNF-α, which is deeply involved in the progression of DN [46,47,48], can induce endothelial glycocalyx dysfunction [13,49,50,51] and increase vascular endothelium permeability [52,53]. Altogether our data indicate that HPSE1 could potentially be involved in the degradation of HS in peritubular capillaries in experimental DN. This may occur either through a direct effect of the enzyme, derived from renal proximal tubules on the microvascular endothelium, or via the induction of TNF-α by interstitial macrophages.

Another important finding of the present study is that treating BTBR *ob*/*ob* mice with ACEi protected the peritubular capillary endothelial glycocalyx by preventing the loss of endothelial sialic acid, and possibly HS, through the inhibition of tubular HPSE1 expression. This is in line with studies that have shown the capability of renin–angiotensin system inhibitors to modulate glomerular HPSE expression and restore HS in experimental models of metabolic syndrome [54] and in adriamycin nephropathy [55,56]. Whether the effect of ACEi on lowering albuminuria, thus reducing the toxic effect of albumin on HPSE stimulation in renal proximal tubules, could contribute to maintaining HS and preserving the peritubular capillary glycocalyx, is a possibility that deserves further investigation. Finally, the anti-inflammatory effects of ACEi, which reduced macrophage infiltration and TNF-α production, could also concur to maintain the integrity of the endothelial superficial layer in diabetic mice.

In conclusion, our results demonstrate that the integrity of the endothelial glycocalyx is essential for preserving the peritubular capillaries and indicate that the endothelial glycocalyx is an important target of the beneficial effects exhibited by ACEi in diabetic mice.

## 4. Materials and Methods

### 4.1. Experimental Design

Male BTBR *ob*/*ob* and BTBR wild-type (WT) mice were obtained from Jackson Laboratories (Bar Harbor, ME, USA). Mice were housed in a temperature-controlled room regulated with a 12:12 h light–dark cycle and allowed free access to standard diet and water. BTBR *ob*/*ob* mice, at 8 weeks of age, when they had already developed albuminuria, were randomised on the basis of urinary albumin-to-creatinine ratio and allocated to 2 experimental groups (n = 7 mice/group) that received either vehicle or lisinopril (30 mg/kg/day, in the drinking water) for 10 weeks until 18 weeks of age. BTBR WT mice (n = 6) were followed-up for the same period and used as the control group. Mice were monitored to assess their body weight and housed in metabolic cages for 24 h urine collection for albuminuria assessment. Blood samples were collected for glucose measurement. At sacrifice, mice were euthanised using CO_2_, and their kidneys were harvested for analyses.

### 4.2. Biochemical and Renal Function Parameters

Blood glucose levels were assessed with a reflectance metre (OneTouch UltraEasy, LifeScan, Milan, Italy). Twenty four-hour urinary albumin excretion was measured using ELISA (Bethyltest kit, E101, A90-134A and A90-134P, Bethyl Laboratories Inc., Montgomery, TX, USA). Urinary creatinine was determined with the Jaffé method using an autoanalyser (DxC800, Beckman Coulter, Rome, Italy). Renal function was assessed by measuring the GFR using iohexol plasma clearance [57].

### 4.3. Systolic Blood Pressure

The systolic blood pressure of conscious mice was assessed using a computerised tail-cuff system (BP-2000 Blood Pressure Analysis System, Visitech System, Apex, White Oak, NC, USA).

### 4.4. Transmission Electron Microscopy Analysis

#### 4.4.1. Ultrastructure of Peritubular Capillaries

Kidney tissue fragments were fixed overnight in 2.5% glutaraldehyde in 0.1 M cacodylate buffer (pH 7.4), washed repeatedly in the same buffer and then fixed in 1% tannic acid and 1% glutaraldehyde in PBS (0.1 M, pH 7.3) for 2 h. After two 15 min rinses in PBS containing 0.1 M sucrose, tissue was post-fixed in 1% OsO_4_, dehydrated with ascending grades of alcohol and embedded in epon resin. Ultra-thin sections were stained with Uranyless and lead citrate (Electron Microscopy Sciences, Hatfield, PA, USA) and examined using a transmission electron microscope (Morgagni 268D, Philips, Brno, Czeck Republic).

Peritubular capillaries were analysed in BTBR WT mice (n = 4) and in BTBR *ob*/*ob* mice that received a vehicle (n = 4) or were treated with ACEi (n = 5). At least 8 images were acquired per mouse. In addition, for each capillary, several images were taken at an original magnification of ×18,000 to enable the reconstruction of the entire capillary profile. Changes in the thickness of endothelial basement membrane of peritubular capillaries were evaluated in the areas where it was clearly seen and distinguished from the tubular basement membrane. The evaluation of denuded basement membrane was performed by examining the images of the entire circumference of the capillary regardless of whether the epithelial basement membrane and the endothelial basement membrane were distinguishable as two entities. For each peritubular capillary, the presence or absence of ultrastructural abnormalities was assessed, and for each mouse. the percentage of peritubular capillaries, showing endothelial or basement membrane changes, was quantified. For each group, data were expressed as mean percentage ± SEM.

#### 4.4.2. Endothelial Glycocalyx

The endothelial glycocalyx was detected using in vivo lanthanum staining [58]. Mice were anaesthetised and perfused with HEPES-buffered mammalian Ringer through a cannula placed in the left ventricle of the heart. An incision was made in the right atrium to allow free blood outflow. Then, a solution of 2% glutaraldehyde, 2% sucrose, and 0.1 M sodium cacodylate buffer (pH 7.3), containing 2% lanthanum nitrate was intracardially perfused. The left kidneys were harvested, and small fragments were fixed in the perfusion solution for 2 h and then immersed overnight in the same solution as above, without glutaraldehyde. The specimens were washed with cacodylate buffer (pH 7.3) and 2% sucrose, post-fixed in 1% osmium OsO_4_, dehydrated using ascending grades of alcohol and embedded in epon resin. Ultra-thin sections were examined using a transmission electron microscope (Morgagni 268D; Philips, Brno, Czech Republic). An average of 10 peritubular capillaries per mouse were examined in BTBR WT mice (n = 3) and in BTBR *ob*/*ob* mice that received a vehicle (n = 4) or were treated with ACEi (n = 5).

### 4.5. Immunohistochemistry

#### 4.5.1. Immunofluorescence Analysis

OCT-frozen kidney sections were fixed with acetone, treated with 1% bovine serum albumin (BSA) to block nonspecific sites, and incubated with a rat anti-mouse CD31 antibody (550274, BD Pharmingen, San José, CA, USA, 1:100), followed by Cy3 conjugated goat anti-rat (1:200; Jackson Immuno-Research Laboratories, Cambridge, UK). Peritubular capillary density was assessed using CD31-positive staining in at least 15 randomly acquired non-overlapping interstitial fields of the cortex per sample (original magnification, X630). Images were processed using the ImageJ/Fiji software (ImageJ; National Institutes of Health, Bethesda, MD, USA, version 2.14.0). Values were expressed as a percentage of the CD31-positive area to the total area of the acquired field.

Kidney tissues were immersed overnight in paraformaldehyde fixative at 4 °C and then were transferred to 30% sucrose and frozen in OCT compound. Double immunostaining for DyLight 488-conjugated lycopersicon esculentum lectin (LEL, DL-1174-1, Vector Laboratories, Burlingame, CA, USA, 1:200) with CD31 was performed. Sections were blocked in 1% BSA plus 5% normal goat serum and incubated with rat anti-mouse CD31 antibody diluted in LEL, followed by Cy3 conjugated goat anti-rat. Nuclei were stained with DAPI and renal structure with Cy5-lens culinaris agglutinin (LCA, DyLight 649; Vector Laboratories). The colocalization area was determined through colour threshold analysis, with the threshold value computed using the Otsu method. PFA4%-fixed sections were incubated with the following: FITC-conjugated Sambucus Nigra Lectin (SNA, FL-1301, Vector Laboratories, 1:200), or rabbit anti-heparanase (HPSE, ab85543, Abcam, Cambridge, UK, 1:150), or rabbit anti-TNF-α (GTX110520, GeneTex, Irvine, CA 92606 USA, 1:200), or rat anti-mouse Mac-2 (clone M3/38, Cedarlane, Burlington, ON, Canada, 1:200) antibodies, followed by Cy3 or FITC-conjugated secondary antibodies.

Double immunostainings for HPSE or TNF-α with Mac-2 were performed. For these dual stainings, antigen retrieval was carried out using a decloaking chamber along with a Rodent decloaker buffer (RD913M, Biocare Medical) or a citrate buffer. CD31, SNA, and heparanase-positive signals were quantified in 10–12 fields in each section (original magnification, X630), and the positive areas were expressed as a percentage of the total area. Images were processed using the ImageJ/Fiji software. Negative control slides were incubated with concentration-matched isotype control rat and rabbit IgG antibodies. Fluorescence was examined using an inverted confocal laser microscope (Leica TCS SP8, Leica Microsystems, Wetzlar, Germany).

#### 4.5.2. Immunoperoxidase Analysis

Duboscq-Brazil-fixed 3 μm paraffin-embedded kidney sections were incubated with Peroxidazed 1 (PX968H, Biocare Medical, Pacheco, CA, USA) to quench endogenous peroxidase, after antigen retrieval in a decloaking chamber with Rodent decloaker buffer. After blocking for 30 min with Rodent Block M (RBM961G, Biocare Medical), sections were incubated for 2 h with rat anti-Mac-2 (1:600) antibody followed by Rat on Mouse HRP-Polymer (RT517, Biocare Medical) for 30 min at room temperature. Staining was visualised using diaminobenzidine (BDB2004H, Biocare Medical) substrate solutions. Slides were counterstained with Mayer’s hematoxylin (MHS80-2.5L, Bio Optica, Milan, Italy), mounted with Eukitt mounting medium (09-00250, Bio Optica) and observed using light microscopy (ApoTome, Axio Imager Z2, Zeiss). Negative controls were obtained by omitting the primary antibody on adjacent sections. The number of Mac-2-positive monocytes/macrophages was quantified in 15–20 tubulointerstitial fields per sample (original magnification, X400) and expressed as the average number of cells per field.

### 4.6. Statistical Analysis

The results were expressed as mean ± SEM. Data analysis was performed using Graph Pad Prism 10.0.3 software (Graph Pad, San Diego, CA, USA). Comparisons were made using one-way ANOVA with Tukey’s multiple comparisons post hoc test. Statistical significance was defined as a *p* value < 0.05.

## Figures and Tables

**Table 1:** Laboratory, systemic and renal function parameters measured in nondiabetic and diabetic mice at 18 weeks of age.

Groups	Body Weight	Blood Glucose	SBP	GFR	uACR
	(g)	(mg/dL)	(mmHg)	(μL/min)	(μg/mg)
BTBR WT	39 ± 2	130 ± 7	101 ± 2	202 ± 39	32 ± 3
	(*n* = *6*)	(*n* = *6*)	(*n* = *6*)	(*n* = *5*)	(*n* = *6*)
BTBR *ob*/*ob*					
vehicle	49 ± 2 *	554 ± 20 **	96 ± 4	612 ± 73 *	861 ± 210 **
	(*n* = *7*)	(*n* = *7*)	(*n* = *7*)	(*n* = *6*)	(*n* = *7*)
ACEi	49 ± 2	576 ± 16	83 ±4°	505 ±95	307 ± 65 °
	(*n* = *7*)	(*n* = *7*)	(*n* = *7*)	(*n* = *6*)	(*n* = *7*)

Data are mean ± SEM. * *p* < 0.01, ** *p* < 0.0001 vs. BTBR WT; ° *p* < 0.05 vs. BTBR *ob*/*ob* + vehicle (one-way ANOVA with Tukey’s post hoc test). ACEi, angiotensin-converting enzyme inhibitor; SBP, systolic blood pressure; GFR, glomerular filtration rate; uACR, urinary albumin-to-creatinine ratio.

## Data Availability

All data generated in the study are presented in the manuscript.
